# Long-term effects on healthcare utilisation among spouses of persons with stroke

**DOI:** 10.1186/s12913-023-10286-0

**Published:** 2023-11-24

**Authors:** Frida Labori, Carl Bonander, Mikael Svensson, Josefine Persson

**Affiliations:** 1 Health Economics and Policy, School of Public Health and Community Medicine, Institute of Medicine, University of Gothenburg, Box 463, Gothenburg, 405 30 Sweden; 2 Department of Pharmaceutical Outcomes and Policy, College of Pharmacy, University of Florida, Gainesville, US

**Keywords:** Spouse, Informal care, Caregiver, Healthcare utilisation, Healthcare utilization, Stroke

## Abstract

**Background:**

Stroke is a common and costly disease affecting the person with stroke and their relatives. If the negative effect on the health of informal caregivers to a person with stroke translates into an increased healthcare consumption has not yet been studied. Further, the importance of including costs and health consequences of informal caregiving in health economic evaluation supporting decision-making is an ongoing discussion. Therefore, this study aims to estimate the long-term effect on healthcare utilisation among spouses of persons with a first-ever stroke.

**Method:**

The study population consists of spouses of persons with first-ever stroke events in 2010–2011 and a reference population matched on age, sex and municipality of residence. We have access to information on healthcare utilisation five years before and five years after the stroke event for the whole study population. Using a difference-in-difference approach, the main analysis estimates the effects on primary and specialist outpatient care visits and days with inpatient care per year. Further, we analyse the healthcare utilisation among spouses depending on the modified Rankin Scale (mRS) of the person with stroke.

**Results:**

Our main analysis indicates that spouses have slightly more days with inpatient care five years after the stroke event than the reference population (p = 0.03). In contrast, spouses have fewer primary and specialist outpatient care visits than the reference population following the stroke event. In the analysis where spouses’ healthcare utilisation is analysed according to the mRS status of the person with stroke, we identify the most notable change in the number of visits to specialist outpatient and days with inpatient care among spouses of persons with mRS 3 (dependency in daily activities).

**Conclusion:**

Our study suggests that being the spouse of a person with stroke has minor effects on healthcare utilisation. Further, healthcare utilisation is most affected among the spouses of persons with stroke and dependency in daily activities (mRS 3). According to our results, it does not seem vital to include spouses of persons with stroke healthcare utilisation in health economic evaluations.

**Supplementary Information:**

The online version contains supplementary material available at 10.1186/s12913-023-10286-0.

## Background

Stroke is one of the leading causes of disability worldwide [1]. In the European Union (EU), the total cost of stroke in 2015 was approximately 45 billion EUR, of which 16 billion EUR was related to informal care [2]. Health economic evaluations of different stroke-related interventions are increasing and influence decision-making. However, there is an ongoing discussion on when and what cost and health consequences of informal caregiving should be included in health economic evaluations. To further support the decision-making on what consequences of informal caregiving are to be included in health economic evaluations, more data is needed on the consequences of stroke on informal caregivers. This includes the effect on the healthcare utilisation of spouses, which may incur additional, hidden costs beyond those related to the person affected by the stroke.

Stroke is a disease that appears without warning signs and can be shocking for the person and relatives. Thus, spouses of persons with stroke enter the role as informal caregivers without any or little time for preparation. Previous research reports that being an informal caregiver of a person with stroke can impact the informal caregiver’s health. On the one hand, the role of an informal caregiver can be a positive experience; for example, Haley et al. [3] report that informal caregivers feel needed and appreciate life more [3]. However, being an informal caregiver can also be a negative experience and affect health adversely. Mental disorders such as anxiety and depression are frequent among informal caregivers [4–6]. In addition to mental disorders, informal caregivers have a higher risk for cardiovascular diseases (CVD) [7]. Further, we have previously reported that spouses of persons with stroke have an increased risk of all-cause mortality during a follow-up period of 5 years compared to their matched controls [8].

A previous study has reported that informal caregivers’ health-related quality of life (HRQoL) decreases with increasing stroke severity, where the stroke severity is measured by the National Institutes of Health Stroke Scale (NIHSS) at the hospital admission [9]. Further, it has been shown that spouses of persons with stroke who are dependent in daily activities have poorer physical, mental and general health [10]. The dependency in daily activities of the person with stroke is often measured by the modified Rankin Scale (mRS), a scale from 0 to 6, where 6 represents death, 3–5 represents dependency in daily activities, and 0–2 represents independence in daily activities [11]. It has previously been reported that the mRS of the person with stroke influences the extent of the informal support provided by the spouse, and spouses of persons with stroke and mRS 3–5 provide the most extensive informal support [12].

There is published literature on healthcare utilisation among self-reported informal caregivers in general or informal caregivers to persons with diseases such as dementia. Overall, the literature on healthcare utilisation among informal caregivers is mixed. Shaffer et al. [13], who investigated informal caregivers in general, reported no difference in time since the last routine healthcare check-up or the total number of healthcare contacts between informal and non-informal caregivers [13]. Further, no differences were reported in the total number of health insurance billings [14]. Kolanowski et al. [15], which report on the healthcare utilisation of spouses of persons with dementia, found no difference in the number of outpatient or inpatient visits. However, spouses had statistical significantly more emergency room visits than the comparison group. Cochrane et al. [16] and Rahman et al. [17] also found that informal caregivers utilised more healthcare resources than non-informal caregivers. We note that existing research on informal caregivers’ healthcare utilisation mostly focuses on caregivers of persons with dementia. It is unclear whether the observed patterns are transferable to informal caregivers of persons with stroke, given that stroke has a more sudden onset than dementia.

On the one hand, one could hypothesise that the stroke event could lead to higher healthcare consumption for spouses due to the adverse health effects reported [4–7] and the increased risk of mortality [8] among informal caregivers. On the other hand, it could also lead to lower healthcare consumption if the spouse does not prioritise their health and delays healthcare visits.

This study aims to assess these hypotheses by studying the effects on healthcare utilisation among spouses of persons with first-ever stroke events up to five years after the stroke. As a secondary aim, we also investigate if the effect on healthcare utilisation is related to the mRS of the person with stroke, given the connection between mRS of the person with stroke and the self-reported health of the spouse.

## Method

### Study population and data sources

This longitudinal study was based on Swedish national and regional registries linked via personal identity numbers [18]. The Swedish Stroke Registry (Riksstroke) was used to identify persons with a stroke event in 2010 or 2011. Riksstroke is a national quality register that contains information on acute stroke, with a coverage of 88% in 2010 [19] and 90.5% in 2011 [20]. Statistics Sweden assisted us with identifying the spouses of each person with a stroke identified in Riksstroke. In Statistics Sweden’s registers, a person can be identified as a spouse if they are married, registered partners, or living in the same household with a joint child (biological or adoptive) [21]. Using registry data from Statistics Sweden, each spouse of a person with a stroke was matched with four reference individuals from the general population who were not identified as spouses of persons with stroke in 2010 or 2011. The reference individuals were matched based on sex, age and the municipality of residence. For both spouses and the reference individuals, we received data on the demographic variables: age, sex, municipality of residence and country of birth from the register of the total population (RTB), annual income, and educational level from the longitudinal integrated database for health insurance and labour market studies (LISA) database. The RTB and LISA register is managed by Statistics Sweden and includes all individuals registered in Sweden [21, 22].

From the National Board of Health and Welfares Patient Register, we received information on inpatient care for spouses and the reference population for five years before and after the stroke onset. The National Board of Health and Welfare patient register contains information such as International Classification of Disease (ICD-10) codes, primary diagnosis and length of hospital stay and has full national coverage [23], with only 1.1% of primary diagnoses missing in 2020 [24].

In Sweden, there is no national register for primary and specialised outpatient care (all healthcare personnel), so applications must be sent to each region. We applied for primary and specialised outpatient care data in four regions known by the authors. One region rejected our application, and from one region, we received data; however, due to the data quality, we had to exclude this region. Finally, we acquired primary and specialised outpatient care data from two regional healthcare registers (Region Skåne and Region Västra Götaland). For a part of the analysis, we included spouses and the reference population if they were residents of Region Skåne or Region Västra Götaland in the year of the stroke event. Out of the 10 million inhabitants in Sweden, approximately 30% are residents in Region Skåne or Region Västra Götaland.

Statistics Sweden managed all linkage between all registries. To protect the privacy of the individuals, the data files were pseudonymized before being delivered to us.

### Measurements

We present spouses’ age and sex at the year of the stroke event, where age is a continuous variable, and sex is categorised as *man* or *woman*. We divided the spouses’ country of birth into three categories: *Sweden, Europe* and *outside of Europe*. Further, we separated educational level into three categories: *less than high school* (less than 9 years), *high school* (12 years of education) and *more than high school* (more than 12 years of education). The mean annual disposable income is the spouses’ mean individual income during the year of the stroke event. Later, the disposable income is divided into four quartiles to present the income distribution among spouses and the reference population. To indicate whether the spouse is a spouse to a stroke survivor, we use information from the Riksstroke registry that captures if the person with stroke is *alive* or *dead at three months post-stroke.*

The outcome measurements under investigation were healthcare utilisation divided into three categories: the number of primary care visits, the number of specialised outpatient care visits, and days with inpatient care per year. Primary care visits include appointments with all healthcare personnel connected to a primary care unit. In contrast, specialised outpatient care refers to visits to a specialist unit at a hospital or a specialised clinic and includes appointments with all healthcare personnel. Inpatient care covers admissions to hospitals in Sweden. We excluded primary, specialised outpatient and inpatient care related to women’s health, such as maternity clinics and obstetrics departments, because these contacts are mostly unrelated to ill health and only apply to women.

### Statistical analysis

We computed descriptive statistics as means with standard deviation (SD) for continuous variables and frequencies with corresponding percentages (%) for categorical variables. The descriptive analyses were conducted separately for outpatient care (primary and specialised outpatient care) and inpatient care.

We applied a difference-in-difference approach to estimate the long-term effect on spouses’ healthcare utilisation after a partner’s stroke event compared to the reference population. Difference-in-differences is a quasi-experimental method that compares the post-to-pre-event difference in means in the exposed group to the corresponding difference in the reference population [25]. An essential assumption of the analysis is that both groups would have followed parallel trends without stroke events [25]. This assumption is untestable but often investigated by inspecting whether the trends in the pre-period are parallel. In our main analysis, the pre-period consists of the year of the stroke event (*t*) and five years before (*t*-1 until *t*-5), and the post-period includes five years following the stroke event (*t* + 1 until *t* + 5).

Under the parallel trends assumption, difference-in-differences estimate the average effect of being a spouse to a person with a stroke event over the five years following the event. The 95% confidence interval (CI) and p-values are presented alongside the average effect.

We conducted all analyses in Stata (version 17.0, Stata, College Station TX, USA). For the difference-in-difference analysis, we used the command *xtdidregress*, specially developed for difference-in-difference analysis using panel data. All standard errors have been adjusted to account for individual-level clustering.

### Analysis according to mRS

We estimated the mRS of the person with stroke by mapping the available variables in Riksstroke to mRS using the algorithm by Eriksson et al. [26]. Our secondary aim is to estimate the effect on spouses’ healthcare consumption depending on the mRS of the person with stroke, we have previously reported that spouses of persons with stroke and mRS 3 reports the lowest health-related quality of life [27]. We argued that this might be because spouses of persons with mRS 3 receive less support, even though the person with stroke still lives at home. Due to this argument, we categorised mRS into three groups: mRS 0–2, mRS 3, and mRS 4–5.

### Sensitivity analysis

We carried out three sensitivity analyses. Firstly, given that parallel trends are essential for the validity of the analysis, we conducted propensity score-weighted analyses where we re-weighted the reference population to match the spouses on the trends and levels of the outcome in the pre-period [28]. To implement the approach, we estimated propensity scores for each outcome variable separately, where each of the pre-period outcomes (t-1 to t-5) was entered as separate independent variables and a spouse-reference group indicator as a dependent variable. Secondly, we investigated how sensitive our main results are to include the year of the stroke event in the pre-period, as we did in the main analysis. Therefore, we included the stroke year as part of the post-period in the second sensitivity analysis. Thirdly, we also conducted the inpatient care analysis on the Region Skåne and Region Västra Götaland sample to check the consistency between the two samples.

## Result

### Descriptive statistics

The study population used to analyse inpatient care nationally consisted of 64,734 individuals (13,049 spouses and 51,685 reference individuals), and the study population for the outpatient care analysis in Region Skåne and Region Västra Götaland consisted of 19,315 individuals (3,891 spouses and 15,424 reference individuals).

We present the demographic information for each group in Table 1. As expected, the spouses and reference population have an equal age and gender distribution due to matching. The groups also have similar educational level, countries of birth, and income distribution (Table 1). Detailed information about the mean healthcare utilisation before and after the stroke event is presented separately for spouses and the reference population in Table 2.

**Table 1:** Descriptive statistics of the study population. Descriptive statistics are presented combined for primary- and specialist outpatient care (outpatient care) in Region Skåne and Region Västra Götaland sample and inpatient care in the total sample

Variable	Outpatient care			Inpatient care	
	Spouse	Reference		Spouse	Reference
Number	3 891 (20%)	15 424 (80%)		13 049 (20%)	51 685 (80%)
Women (%)	2 453 (63%)	9 726 (63%)		8 438 (65%)	33 458 (65%)
Age 2011 (SD)	71 (12)	71 (12)		71 (12)	71 (12)
Country of birth					
Sweden (%)	3 341 (86%)	13 416 (87%)		11 324 (87%)	45 208 (87%)
Europe (%)	425 (11%)	1 501 (10%)		1 263 (10%)	4 756 (9%)
Outside Europe (%)	125 (3%)	507 (3%)		462 (3%)	1 721 (3%)
Educational level (%)					
Less than high school	1 633 (42%)	6 025 (39%)		5 096 (39%)	19 036 (37%)
High school	1 467 (38%)	5 869 (38%)		5 091 (39%)	20 151 (39%)
More than high school	791 (20%)	3 530 (23%)		2 862 (22%)	12 498 (24%)
Mean disposable income year of the stroke event (SD)	179 959 SEK (230 751)	185 765 SEK (307 865)		177 655 SEK (188 781)	188 379 SEK (286 931)
Disposable income					
First quartile (%)	1 015 (26%)	3 814 (25%)		3 477 (27%)	12 742 (25%)
Second quartile (%)	967 (25%)	3 864 (25%)		3 254 (25%)	12 897 (25%)
Third quartile (%)	976 (25%)	3 854 (25%)		3 307 (25%)	12 894 (25%)
Fourth quartile (%)	932 (24%)	3 892 (25%)		3 010 (23%)	13 152 (25%)
Person with stroke					
Alive at 3 months	3 843 (99%)	NA		12 890 (99%)	NA
Dead at 3 months	48 (1%)	NA		159 (1%)	NA
Deceased					
2012 (%)	131 (3.4%)	500 (3.2%)		446 (3.4%)	1 559 (3.0%)
2013 (%)	138 (3.7%)	503 (3.4%)		436 (3.5%)	1 546 (3.1%)
2014 (%)	140 (3.9%)	483 (3.3%)		412 (3.4%)	1 596 (3.3%)
2015 (%)	145 (4.2%)	509 (3.7%)		458 (3.9%)	1 723 (3.7%)
2016 (%)	139 (4.2%)	526 (3.9%)		440 (3.9%)	1 673 (3.7%)

**Table 2:** Mean healthcare utilisation before and after the year of the stroke event

	Spouses		Reference population	
	Mean before stroke event (SD)	Mean after stroke event (SD)	Mean before stroke event (SD)	Mean after stroke event (SD)
**Main analysis**				
Primary care (visits)	5.81 (9.6)	8.75 (11.9)	5.59 (8.8)	8.73 (11.7)
Specialised outpatient care (visits)	3.12 (7.5)	3.46 (7.7)	3.12 (7.1)	3.58 (8.1)
Inpatient care (days)	1.01 (5.3)	1.60 (6.8)	0.89 (4.9)	1.47 (6.5)
**mRS categories**				
***Primary care***				
mRS 0–2	5.42 (9.1)	8.4 (11.2)	5.32 (8.6)	8.44 (11.5)
mRS 3	6.57 (9.6)	10.07 (13.6)	6.02 (8.6)	9.65 (12.4)
mRS 4–5	6.56 (10.3)	9.66 (12.2)	6.41 (9.9)	9.90 (12.5)
***Specialised outpatient care***				
mRS 0–2	2.97 (7.5)	3.45 (8.1)	2.96 (7.0)	3.47 (7.8)
mRS 3	3.52 (6.6)	3.52 (6.2)	3.46 (7.6)	3.82 (8.7)
mRS 4–5	3.77 (9.7)	3.77 (7.7)	3.51 (7.3)	3.85 (7.7)
***Inpatient care***				
mRS 0–2	0.84 (4.9)	1.30 (6.1)	0.78 (4.6)	1.28 (6.1)
mRS 3	1.42 (5.9)	2.29 (7.8)	1.15 (5.6)	1.97 (7.5)
mRS 4–5	1.33 (6.2)	2.15 (7.5)	1.12 (5.4)	1.94 (7.1)

### Main analysis

**Fig. 1:**
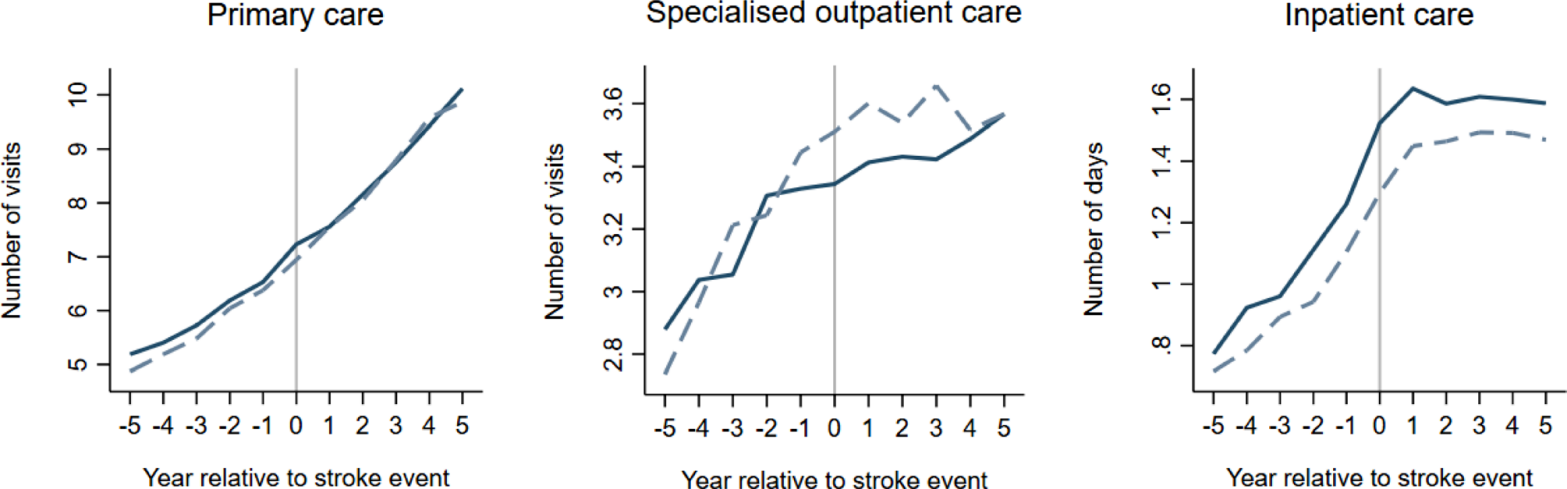
Illustration of the results from the main analysis. The solid line represents spouses, and the dashed line represents the reference population

Our difference-in-differences analysis suggests that spouses of persons with stroke have an average of 0.088 additional days with inpatient care in the five years following the stroke event compared to the reference population (Fig. 1; Table 3). In contrast, spouses of persons with stroke appeared to have fewer primary and specialised outpatient care visits (Fig. 1). However, the estimates (primary and specialised outpatient care) are not statistically significant, and the change is small in relative terms (Table 3).

### Analysis according to mRS

**Fig. 2:**
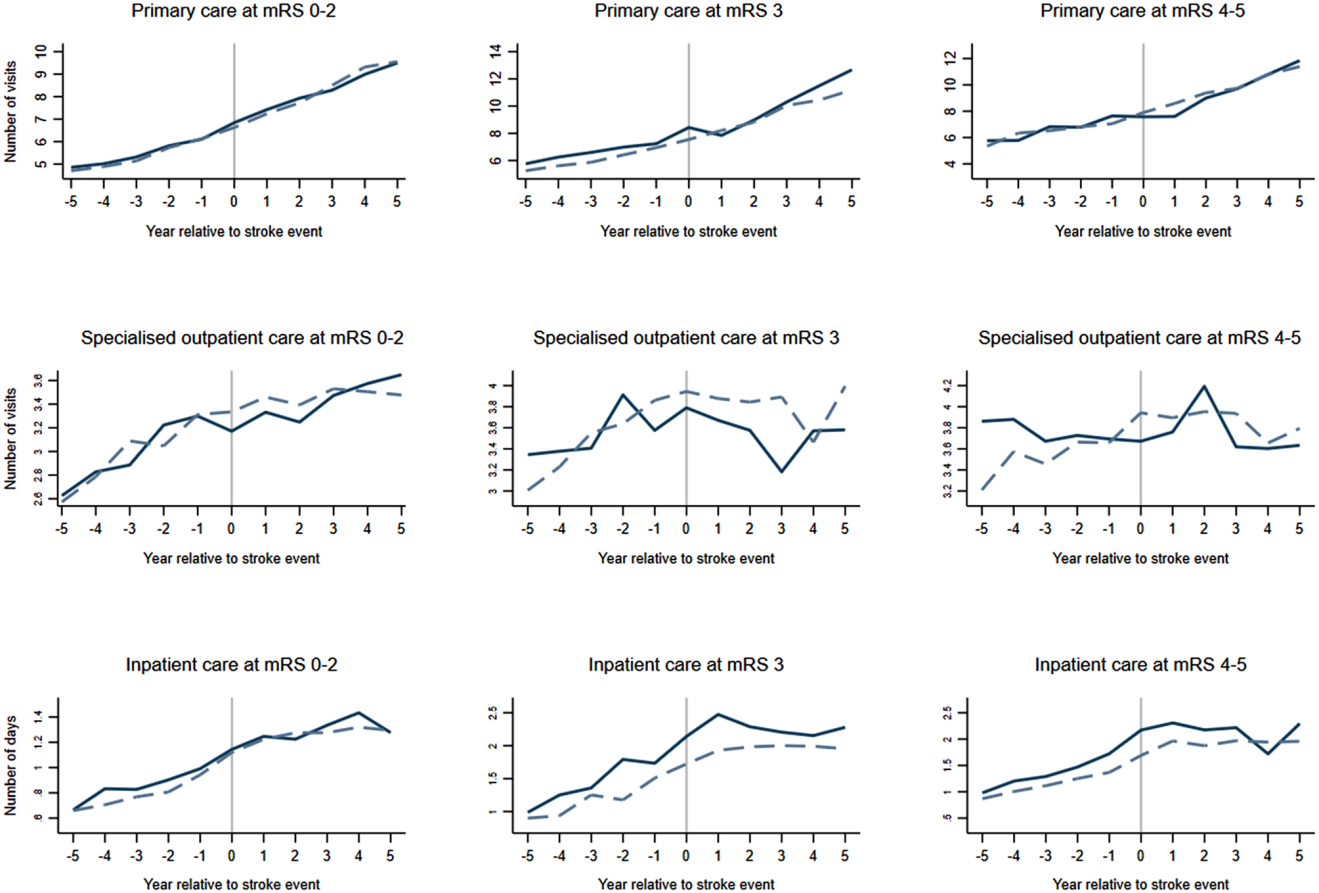
Illustration of the results from the analysis based on mRS of the person with stroke. The solid line represents spouses, and the dashed line represents the reference population

The results of the difference-in-differences analysis stratified by mRS are reported in Table 3 and illustrated in Fig. 2. None of the stratified estimates was statistically significant. However, there was an indication that spouses’ healthcare consumption is particularly affected if being a spouse to a person with mRS 3 after a stroke. Specifically, in the number of visits to specialised outpatient care, the point estimates suggested a decrease of 7.7% (relative change) and a 7.7% increase in the number of days with inpatient care among spouses of persons with mRS 3. We did not find a similar pattern regarding the number of visits to primary care, where we noted the most considerable relative change among spouses of persons with stroke and mRS 4–5 (Table 3).

**Table 3:** Results from the main analysis, analysis based on mRS category and sensitivity analysis

Variable	N observations	Coefficient (95% CI)	p-value	Relative change
**Main analysis**				
Primary care (visits)	19 315	-0.129 (-0.420; 0.161)	0.383	0.985
Specialised outpatient care (visits)	19 315	-0.040 (-0.252; 0.171)	0.708	0.988
Inpatient care (days)	64 734	0.088 (0.009; 0.168)	0.030*	1.058
**mRS categories**				
***Primary care (visits)***				
mRS 0–2	11 925	-0.071 (-0.415; 0.273)	0.687	0.992
mRS 3	2 416	-0.281 (-1.300; 0.738)	0.589	0.973
mRS 4–5	2 498	-0.501 (-1.329; 0.329)	0.235	0.951
***Specialised outpatient care (visits)***				
mRS 0–2	11 925	0.017 (-0.263; 0.298)	0.904	1.005
mRS 3	2 416	-0.294 (-0.823; 0.235)	0.275	0.923
mRS 4–5	2 498	0.015 (-0.592; 0.622)	0.962	1.004
***Inpatient care (days)***				
mRS 0–2	39 870	0.003 (-0.091; 0.096)	0.957	1.002
mRS 3	7 217	0.164 (-0.124; 0.452)	0.265	1.077
mRS 4–5	9 114	0.124 (-0.109; 0.357)	0.296	1.061
**Sensitivity analysis**				
***Year of stroke event in the post-period***				
Primary care (visits)	19 315	-0.081 (-0.360; 0.198)	0.569	NA
Specialised outpatient care (visits)	19 315	-0.087 (-0.290; 0.115)	0.399	NA
Inpatient care (days)	64 734	0.107 (0.026; 0.187)	0.009*	NA

*Statistically significant at a 5% significance level

### Sensitivity analysis

The results were comparable to those from the main analysis when we included the year of the stroke event in the post-period in the sensitivity analysis (Table 3). The results from the propensity score-weighted analyses were also similar to the main analysis (Additional file 1; Additional file 2) as well as the analysis according to mRS categories (Additional file 2; Additional file 3), which provides suggestive evidence that the main results are not sensitive to violations of the parallel trends assumption. Finally, the results did not differ when we tested the consistency between the samples by carrying out the inpatient care analysis on the Region Skåne and Region Västra Götaland sample (Additional file 4).

## Discussion

Our study aimed to investigate if healthcare utilisation changes among spouses of persons with stroke after the stroke event compared to a reference population. Generally, the impact on spouses’ healthcare utilisation seems small. However, we identified some changes, including a small but statistically significant increase in the number of days with inpatient care. The relative change in the days with inpatient care is 5.8%, and in the absences of the stroke event, it is estimated that spouses would have 1.5 days with inpatient care instead of 1.6 days with inpatient care.

Our study contributes to the literature on healthcare utilisation among informal caregivers by being, to our knowledge, the first study to investigate the effects of being a spouse of a person with a first-ever stroke on healthcare utilisation. Our results somewhat support the results of Shaffer and Nightingale [13], who studied healthcare consumption among self-reported informal caregivers in the United States and Baumgarten et al. [14], who studied elderly with dementias healthcare consumption, where none of the studies found any statistically significant difference regarding healthcare utilisation.

One aspect that could affect that we do not identify any large changes in the overall results among spouses of persons with stroke could be the generous healthcare system in Sweden. Sweden is one of the countries with the most formal care measured by an index including, for example, long-term beds per 1,000 population 65 or older [29]. The fact that Sweden has many long-term beds could lower the burden on informal caregivers, which is essential to consider when interpreting these results and transferring them to other contexts with different healthcare systems.

In our analysis based on mRS, we found suggestive evidence that the largest effect on specialised outpatient and inpatient care is among spouses of persons with mRS 3. These results should be interpreted cautiously, as they are not statistically significant and have wide confidence intervals. Nevertheless, these findings align with our previous arguments, i.e., that spouses of persons with stroke and mRS 3–5 are most affected. Specifically, there might be a heavier burden on spouses of persons with stroke and mRS 3 as they usually are considered dependent in daily activities and still live at home with homecare services. In contrast, persons with stroke and mRS 4–5 often live in special housing. Future studies with larger samples should consider investigating subgroups based on mRS and the potential importance of including spouses of persons with stroke and mRS 3–5 healthcare utilisation in health economic evaluation.

Overall, our findings indicate that spouses of persons with stroke do not significantly change their healthcare utilisation, suggesting that the consequence of leaving this aspect out of health economic evaluations should be limited. However, there might be other effects, such as time spent on informal caregiving and loss of income for the spouse of a person with a stroke, where inclusion might be crucial for the validity of health economic evaluations. Additionally, the size of the extended family, including the number of adult children, may influence the size of the effect on spousal caregivers. Future research should consider investigating the importance of including these aspects.

### Strengths and limitations

One of the strengths of our study is that it is based on extensive, high-quality register data. Our study population is a national sample of spouses of persons with stroke in 2010 and 2011 in Sweden. In addition, our study period covered eleven years (five years before and after the stroke event), which allowed us to study long-term impacts and handle unobserved, time-invariant confounding by applying a difference-in-differences design. We received high-quality data on inpatient care from The national patient register, which has full national coverage [23], and only approximately 1% of primary diagnoses are missing [24]. Unfortunately, Sweden has no national register for primary and specialised outpatient care (visits to all healthcare personnel). Therefore, we could only get information on primary and specialised outpatient care from two large regions in Sweden, which may limit the generalisability of these results.

For a few of the outcomes, such as days with inpatient care, it looks like spouses of persons with stroke have higher healthcare consumption already before the stroke event (Fig. 1), which could be of concern. However, one of the strengths when using the difference-in-difference method is that this possible concern is automatically handled in the first difference of the difference-in-difference equation as long as the parallel trends assumption holds.

While our results suggest a limited impact, it would have been helpful to have the actual cost for each healthcare visit since the cost per visit and day with inpatient care varies depending on the type of visit. Unfortunately, it was impossible to calculate the actual cost for each healthcare visit due to limitations in the data material we received. We, therefore, focused on the number of healthcare visits.

## Conclusions

Our study indicates that spouses’ healthcare consumption is not affected considerably after the stroke event of the person with stroke. Overall, it does not seem crucial to include the healthcare utilisation of spouses of persons with stroke in health economic evaluations relating to stroke.

### Electronic supplementary material

Below is the link to the electronic supplementary material.

Supplementary Material 1

Supplementary Material 2

Supplementary Material 3

Supplementary Material 4

## Data Availability

The datasets used and/or analysed during the current study are available from the corresponding author upon reasonable request.

## List of abbreviations

CI: Confidence interval
CVD: Cardiovascular diseases
EU: European Union
ICD-10: The International Classification of Diseases, 10th Revision
LISA: the longitudinal integrated database for health insurance and labour market studies
mRS: Modified Ranking Scale
Riksstroke: The Swedish stroke registry
RTB: Register of the total population
SD: Standard Deviation
TIA: Transient ischemic attack
