# Supplementary material for: Long-term effects on healthcare utilisation among spouses of persons with stroke
Source: BMC Health Serv Res. 2023 Nov 24;23:1298. doi: 10.1186/s12913-023-10286-0 (PMC10675871; doi:10.1186/s12913-023-10286-0)
Supplement: Supplementary file 1 — Supplementary Material 1 [file 12913_2023_10286_MOESM1_ESM.docx]

**Additional file 1**

**Table**. Main analysis and analysis based on mRS and sensitivity analysis, weighted with propensity scores.

| **Variable** | **N observation** | **Propensity score-weighted coefficient (95% CI)** | **p-value** | **Relative change** |
| --- | --- | --- | --- | --- |
| **Main analysis** |  |  |  |  |
| Primary care (visits) | 19 315 | -0.074 (-0.367; 0.219) | 0.621 | 0.992 |
| Specialised outpatient care (visits) | 19 315 | -0.030 (-0.242; 0.182) | 0.781 | 0.991 |
| Inpatient care (days) | 64 734 | 0.129 (0.048; 0.210) | 0.002* | 1.088 |
|  |  |  |  |  |
| **Analysis based on mRS categories** |  |  |  |  |
| ***Primary care (visits)*** |  |  |  |  |
| mRS 0-2 | 11 925 | -0.032 (-0.378; 0.313) | 0.855 | 0.996 |
| mRS 3 | 2 416 | -0.046 (-1.100; 1.009) | 0.932 | 0.995 |
| mRS 4-5 | 2 498 | -0.437 (-1.310; 0.436) | 0.327 | 0.957 |
|  |  |  |  |  |
| ***Specialised outpatient care (visits)*** |  |  |  |  |
| mRS 0-2 | 11 925 | 0.003 (-0.279; 0.285) | 0.983 | 1.001 |
| mRS 3 | 2 416 | -0.224 (0.755; 0.306) | 0.407 | 0.940 |
| *mRS 4-5* | 2 498 | 0.102 (-0.521; 0.725) | 0.749 | 1.028 |
|  |  |  |  |  |
| ***Inpatient care (days)*** |  |  |  |  |
| mRS 0-2 | 39 870 | 0.027 (-0.068; 0.122) | 0.574 | 1.021 |
| mRS 3 | 7 217 | 0.244 (-0.051; 0.540) | 0.105 | 1.119 |
| mRS 4-5 | 9 114 | 0.202 (-0.037; 0.440) | 0.097 | 1.104 |
|  |  |  |  |  |
| **Sensitivity analysis** |  |  |  |  |
| ***Year of stroke year included in post-period*** |  |  |  |  |
| Primary care (visits) | 19 315 | -0.017 (-0.299; 0.265) | 0.906 | NA |
| Specialised outpatient care (visits) | 19 315 | -0.063 (-0.266; 0.140) | 0.545 | NA |
| Inpatient care (days) | 64 734 | 0.159 (0.077; 0.241) | <0.001* | NA |

* Statistically significant at a 5% significance level
