# Supplementary material for: Long-term effects on healthcare utilisation among spouses of persons with stroke
Source: BMC Health Serv Res. 2023 Nov 24;23:1298. doi: 10.1186/s12913-023-10286-0 (PMC10675871; doi:10.1186/s12913-023-10286-0)
Supplement: Supplementary file 2 — Supplementary Material 2 [file 12913_2023_10286_MOESM2_ESM.docx]

**Additional file 2**

**Figure**. Illustration of the results from the propensity score-weighted analysis on the main outcomes.


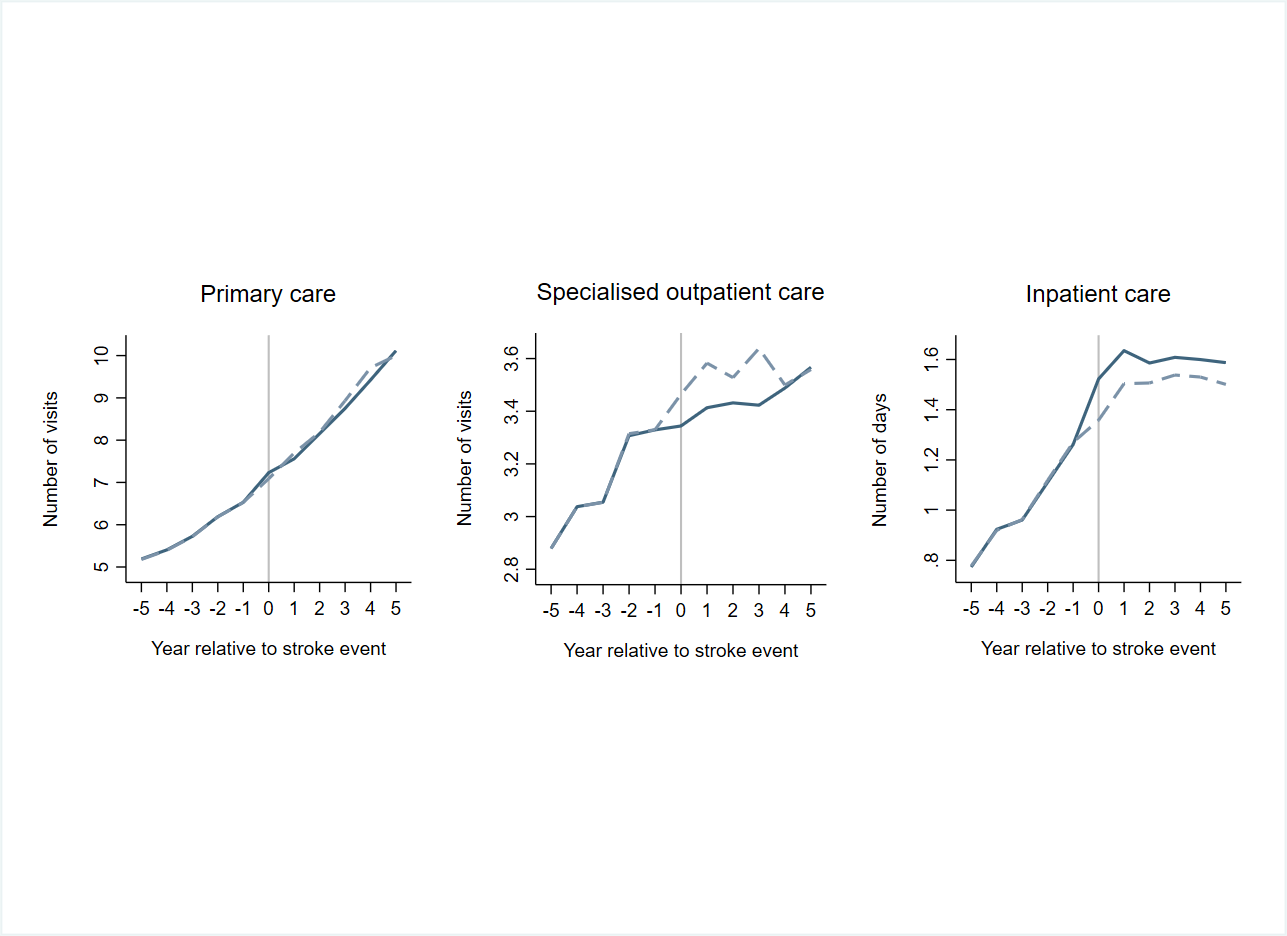


The solid line represents spouses, while the dashed line represents the reference population.
