# Supplementary material for: Long-term effects on healthcare utilisation among spouses of persons with stroke
Source: BMC Health Serv Res. 2023 Nov 24;23:1298. doi: 10.1186/s12913-023-10286-0 (PMC10675871; doi:10.1186/s12913-023-10286-0)
Supplement: Supplementary file 3 — Supplementary Material 3 [file 12913_2023_10286_MOESM3_ESM.docx]

**Additional file 3**

**Figure**. The figure illustrates the results from the propensity score-weighted analysis based on mRS.


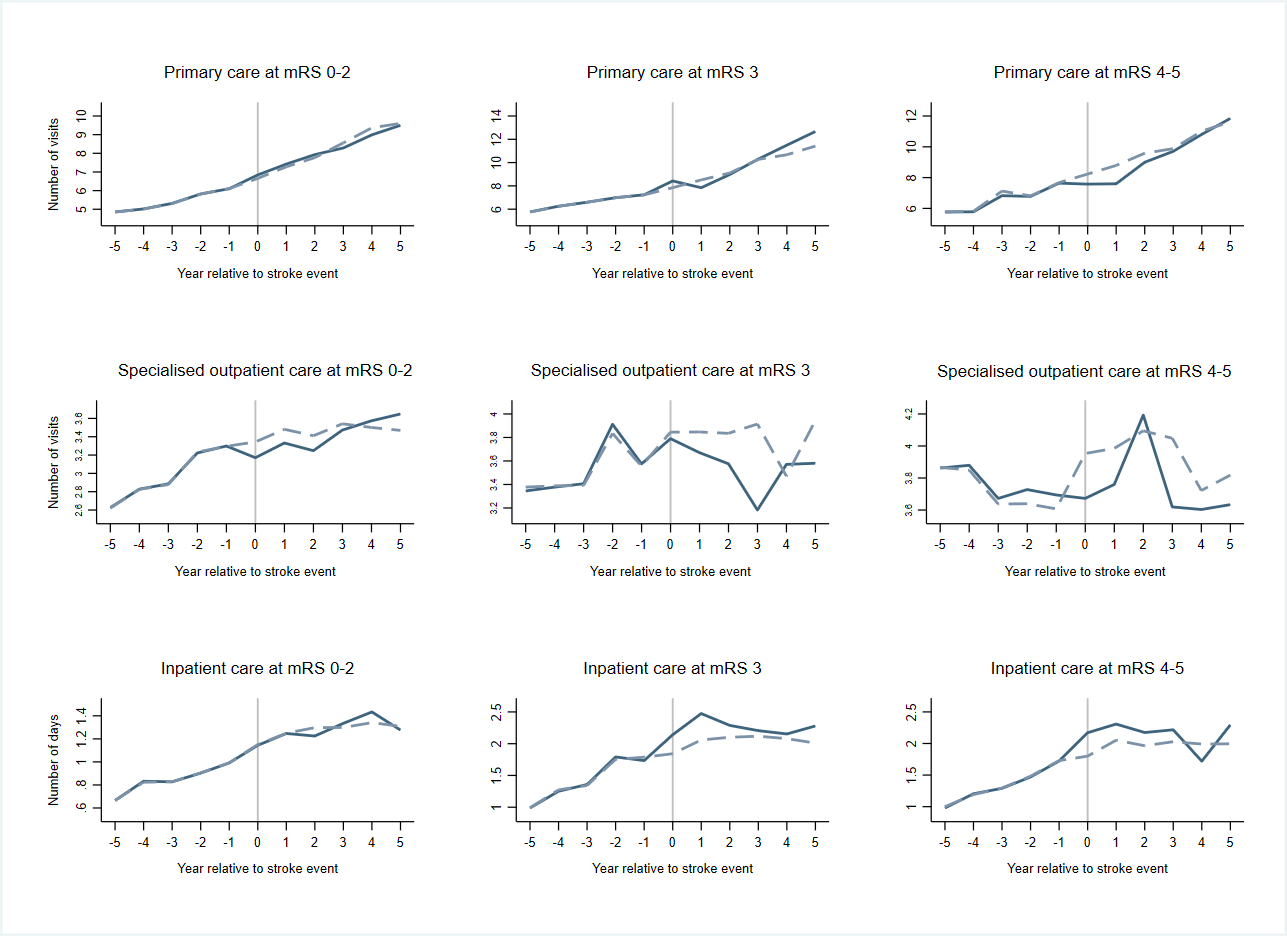


The solid line represents spouses, while the dashed line represents the reference population.
