# Supplementary material for: Long-term effects on healthcare utilisation among spouses of persons with stroke
Source: BMC Health Serv Res. 2023 Nov 24;23:1298. doi: 10.1186/s12913-023-10286-0 (PMC10675871; doi:10.1186/s12913-023-10286-0)
Supplement: Supplementary file 4 — Supplementary Material 4 [file 12913_2023_10286_MOESM4_ESM.docx]

**Additional file 4**

**Table**. Sensitivity analysis, the effect on inpatient care in the sample from Region Skåne and Region
Västra Götaland

|  | **N observations** | **Unweighted coefficient (95% CI)** | **p-value** |
| --- | --- | --- | --- |
| Inpatient care (days) | 19 315 | 0.125 (-0.021; 0.271) | 0.093 |
| Inpatient care days at mRS 0-2 | 11 925 | -0.023 (-0.196; 0.149) | 0.792 |
| Inpatient care days at mRS 3 | 2 416 | 0.237 (-0.243; 0.716) | 0.333 |
| Inpatient care days at mRS 4-5 | 2 498 | 0.298 (-0.155; 0.752) | 0.197 |
